# Atypical Sensory Loss Pattern in an Isolated Thalamic Stroke: A Case Report

**DOI:** 10.7759/cureus.71607

**Published:** 2024-10-16

**Authors:** Mariam Tadros, David Phrathep, Emily Lewandowski, Marc R Mohammed, Tristan Tinney, Zachary Abdo, Christopher Kline

**Affiliations:** 1 Department of Physical Medicine and Rehabilitation, Lake Erie College of Osteopathic Medicine, Bradenton, USA; 2 Department of Physical Medicine and Rehabilitation, Brooks Rehabilitation Hospital, Jacksonville, USA; 3 Department of Physical Medicine and Rehabilitation, Mayo Clinic Alix School of Medicine, Jacksonville, USA; 4 Department of Internal Medicine, Touro College of Osteopathic Medicine, New York, USA; 5 Department of Emergency Medicine, Ascension St. Vincent's Southside Hospital, Jacksonville, USA

**Keywords:** atypical thalamic stroke, cerebrovascular accident, computed tomography imaging, ischemic stroke, magnetic resonance imaging, thalamic stroke

## Abstract

This case report discusses a 51-year-old male who presented to the emergency department (ED) with left-sided hemiparesthesia and left leg incoordination. The initial brain computed tomography (CT) scan was negative, and the follow-up brain CT three days after the onset of symptoms was also negative. Although sensitivity and specificity are not 100%, CT remains the first-line diagnostic test for detecting a cerebrovascular accident (CVA). In this unique case, CT was not sufficient. Following two negative CT scans, magnetic resonance imaging (MRI) finally revealed the cause of this patient's symptoms, an ischemic incident in the right thalamus. Thalamic strokes typically present with contralateral hemiparesis and hemisensory loss, unreactive pupils, and gaze palsy with gaze deviation away from the side of the infarct. It is unusual to see a thalamic lesion present with pure hemiparesthesia without facial involvement. This patient's clinical presentation is discussed, as well as future investigations and ways to prevent this diagnostic delay. This case demonstrates the importance of follow-up imaging based on the clinical presentation of potentially subtle imaging findings.

## Introduction

Stroke is one of the leading causes of morbidity and mortality worldwide, with ischemic stroke accounting for approximately 62% of all cases [1]. Early detection and prompt treatment are critical for improving outcomes and reducing the risk of long-term disability. Computed tomography (CT) of the brain is typically the first-line imaging modality used in the emergency department (ED) to diagnose cerebrovascular accidents (CVAs) due to its wide availability, rapid acquisition, and ability to quickly rule out hemorrhagic stroke [2]. However, the sensitivity of CT for detecting early ischemic changes, particularly in specific brain regions, can be limited [3].

The thalamus is a deep brain structure that plays a crucial role in sensory and motor signal relays and the regulation of consciousness. Strokes affecting the thalamus are uncommon and can present with a wide range of symptoms depending on the specific vascular territory involved. Thalamic strokes often result in contralateral hemiparesis, hemisensory loss, and, in some cases, altered consciousness, ocular movement abnormalities, facial muscle weakness, slurred speech, and loss of balance [4]. The variability in clinical presentation and the limitations of initial imaging studies can sometimes lead to delays in diagnosis, which may affect patient management and outcomes [5]. This case highlights the limitations of CT in detecting certain ischemic events and underscores the importance of considering advanced imaging modalities when clinical suspicion remains high despite initial negative findings. 

## Case presentation

A 51-year-old Caucasian male with a past medical history of hypertension, chronic back pain, gastroesophageal reflux disease, and alcohol abuse presented to the community ED with complaints of left-sided hemiparesthesia with subsequent left leg incoordination. He stated that his symptoms started three days ago while he was traveling abroad to Spain on a 14-day cruise. The symptoms were so severe that he had checked himself into the ED in Spain, where a CT scan of the brain was completed and reported as negative for any acute findings. Because symptoms persisted, he returned the next day by plane to the United States and presented to the ED with worsening symptoms. He described the paresthesias as sudden in acuity and moderate in severity throughout his left arm and leg. He mentioned that his paresthesias were somewhat debilitating to his activities of daily living (ADLs). He was unable to identify a specific aggravating or relieving factor to his symptoms. He had no other associated symptoms. He denied fever, headache, vision changes, musculoskeletal weakness, and changes in urination and bowel movements. Upon arrival at the ED, his vitals were unremarkable, except for a high blood pressure of 158/106. His electrocardiogram showed no abnormalities, and his blood glucose levels were within the normal limits. Other labs were unremarkable, except for a slightly elevated prothrombin time (PT) of 14.3 seconds, international normalized ratio (INR) of 1.3, and partial thromboplastin time (PTT) of 31.7 seconds (Table 1).

**Table 1:** Patient lab values This table is adapted from the American Board of Internal Medicine [6].

Hematologic	Patient value	Reference range
White blood cell count	6.9 k/mcL	4.5-11.0 k/mcL
Red blood cell count	4.7 million/mcL	4.3-5.9 million/mcL
Hemoglobin	13.7 g/dL	13.5-17.5 g/dL
Hematocrit	40.9%	41%-53%
Mean corpuscular volume	87.2 fL	80-100 fL
Mean corpuscular hemoglobin concentration	33.5 Hb/cell	31%-36% Hb/cell
Platelet count	279 k/mcL	150-400 k/mcL
Neutrophils	66 %	54%–62%
Lymphocytes	24%	25%–33%
Monocytes	9%	3%–7%
Eosinophils	1%	1%–3%
Basophils	1%	0%–1%
Coagulation	Patient value	Reference range
PT	14.3 seconds	11–15 seconds
PTT	31.7 seconds	25–40 seconds
INR	1.3	0.9–1.3
General chemistry	Patient value	Reference range
Sodium	143 mmol/L	136–146 mmol/L
Potassium	3.4 mmol/L	3.5–5.0 mmol/L
Chloride	108 mmol/L	95–105 mmol/L
Bicarbonate	26 mmol/L	22–28 mmol/L
Blood urea nitrogen	21 mg/dL	7–18 mg/dL
Creatinine	0.8 mg/dL	0.6–1.2 mg/dL
Glucose (fasting)	87 mg/dL	70–100 mg/dL
Hepatic	Patient value	Reference range
Bilirubin, total/direct	0.8 mg/dL	0.1–1.0 mg/dL
Alkaline phosphatase	79 U/L	25–100 U/L
Aspartate aminotransferase	25 U/L	12–38 U/L
Alanine aminotransferase	34 U/L	10–40 U/L
Proteins, total	7.3 g/dL	6.0–7.8 g/dL
Albumin	4.4 g/dL	3.5–5.5 g/dL
Other, hematologic	Patient value	Reference range
Troponin I	<0.01 ng/mL	≤0.04 ng/mL

**Figure 1:**
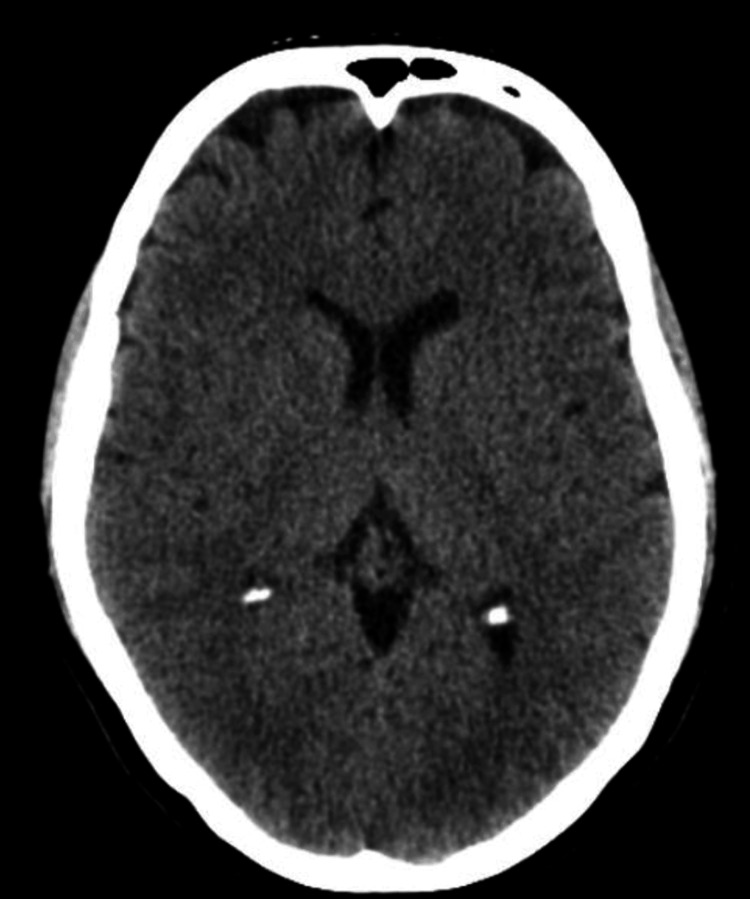
CT of the brain without contrast Initial negative brain CT showing no evidence of vessel occlusion, hemorrhage, or mass effect.

With suspicion of stroke, a CT of the brain, head, and neck was ordered and returned back all negative with no evidence of intracranial hemorrhage, stenosis, or mass effect (Figure 1). This was the second time CT imaging of the brain was negative for acute stroke since the initial onset of the patient’s symptoms. Once stroke was ruled out, a CT of the lumbar spine was ordered and was also negative for significant pathology. By ruling out spinal cord causes of the patient’s symptoms, a neurologist was consulted for neurological assessments and noted left-sided hemiparesthesia and an NIH Stroke Scale/Score of 1. The neurologist appeared to have seen a possible spot on the CT of the brain concerning a CVA not detected by a radiologist and recommended aspirin 324 mg and Plavix (clopidogrel) 300 mg as well as continuation of the patient's current regimen of hydrochlorothiazide for hypertension. The team deemed the patient not an alteplase candidate but that he will be admitted for further evaluation and confirmation of suspicion.

On admission to the hospital, the patient was started on one dose of aspirin 325 mg and one dose of Plavix (clopidogrel) 300 mg. His vital signs were a temperature of 36.9 °C, heart rate of 69 beats per minute, respiratory rate of 16, blood pressure of 130/85, and oxygen saturation of 98%. The general assessment revealed a patient who was comfortable, lying in bed, but actively participating in conversation. Examination of the eyes indicated normal findings with equal, round pupils that reacted to light and intact extraocular movements. The neck was supple and nontender, with no thyroid gland enlargement. Respiratory examination revealed clear lung fields, nonlabored breathing, and equal breath sounds without chest wall tenderness. Cardiovascular examination showed a normal heart rate and rhythm, with clear heart sounds and good peripheral pulses without murmurs or edema. Musculoskeletal examination demonstrated normal strength in all four extremities. The integumentary system appeared normal, with warm skin and no evidence of pallor or rash. The neurological assessment indicated an alert individual who was oriented to person, place, time, and situation but presented with continued left-sided paresthesia on sensation testing in the upper and lower extremities. He presented with no nystagmus, eye closure and smile were intact, no dysarthria noted, and all other cranial nerves were intact. Finger-to-nose and heel-to-shin testings were unremarkable. Psychiatric evaluation revealed a cooperative patient with an appropriate mood and affect. 

**Figure 2:**
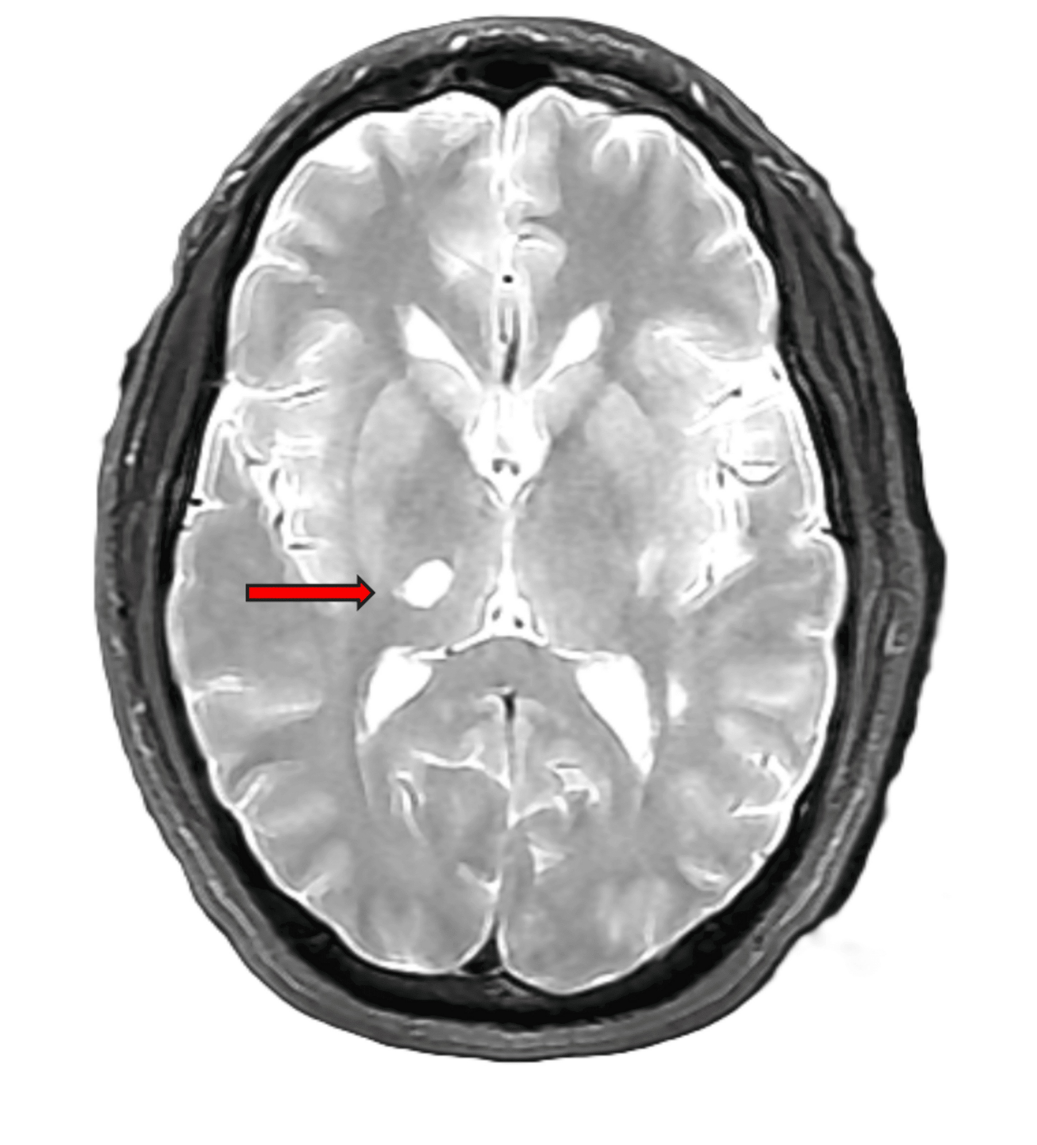
Axial T2 MRI of the brain without contrast

Due to continued paresthesia, MRI of the brain without contrast was ordered for further stroke evaluation. On MRI of the brain without contrast, the reading radiologist found mild white matter abnormalities which likely reflected mild chronic small vessel ischemic disease and an acute infarct in the right thalamus without evidence of acute hemorrhage (Figure 2).

The neurologist concluded the cause of the patient’s symptoms was due to a right thalamic infarct. The patient underwent a bubble study that was unremarkable, which ruled out a cardioembolic cause. The speech therapy assessment revealed that the patient's swallowing function was within functional limits for liquids and solids, with no need for intervention indicated. The patient tolerated thin liquids, puree, and regular solid trials without any signs of oropharyngeal dysphagia. Recommendations included continuing with a regular/thin liquid diet and standard safe swallow precautions. The physical therapist noted the patient's prior functional status as independent in ADLs, mobility, and cognitive-communication skills. Strength and mobility assessments indicated normal strength and range of motion in both lower extremities, with some deviations noted in gait and sensation impairment on the left side. The occupational therapy assessment highlighted the patient's participation in exercises and safety techniques to maintain independence in ADLs, particularly focusing on enhancing sensation and proprioception in the left upper extremity. The patient was noted to be alert and cooperative, with a slight decrease in sensation on the left side without facial involvement. Both physical and occupational therapists emphasized the patient's independence in ADLs and mobility within the home environment, with no further need for skilled therapy services anticipated upon discharge. Vital signs remained stable throughout the assessments, with the patient's cognitive-communication skills intact. Overall, the interdisciplinary team noted the patient's progress and independence in various aspects of functional living, with ongoing support and monitoring provided as needed. 

During the admission, it was recommended that the patient was to continue on aspirin 324 mg and Plavix (clopidogrel) 300 mg for 90 days and then continue with aspirin 81 mg indefinitely. Over the three-day hospital course, the patient’s left-sided paresthesias improved, and he was stable for discharge.

## Discussion

This patient presented with pure left-sided hemiparesthesia without facial involvement, no other focal neurological deficits, and persistent neurologic symptoms after several days, highlighting the challenges and intricacies of diagnosing subtle cerebrovascular events. Hemiparesthesia, characterized by numbness or tingling on one side of the body, often raises concerns for a potential stroke, yet its isolated presentation can complicate initial evaluations [7]. This case highlights the importance of using MRI in a patient with persistent symptoms of stroke despite having two negative CTs and maintaining high levels of suspicion after negative initial tests. Rapid identification and treatment of a stroke can significantly reduce brain damage and improve outcomes [8]. Delays in diagnosis and treatment can lead to irreversible brain damage and increased disability, creating life-or-death situations that can be easily overlooked when having negative initial testing [8].

Typically, noncontrast CT is the primary initial diagnostic test to reveal acute, subacute, and chronic stroke within 24 hours to weeks since symptom onset, respectively [9]. This imaging modality has the ability to identify the location of infarction as well as the ability to exclude other causes of neurologic dysfunction, such as hemorrhage or space-occupying lesions. In this case, within 72 hours from the onset of acute stroke symptoms, this patient had a negative CT angiography of the brain, head, and neck with and without contrast, a negative CT of the head and brain without contrast, and a negative CT of the lumbar spine without contrast. Previous research shows that acute stroke should be visualized on head CT within 24 hours, revealing acute ischemic changes such as edema, loss of white matter and gray matter interface (differentiation), and effacement of the cortical sulci [9]. Location and blood supply of the stroke also play a role when identifying limitations surrounding the use of CT. For example, a middle cerebral artery territory infarction can typically be detected within six hours in 60%-70% of patients, while those within the deep gray matter nuclei can be seen within one hour [10]. Additionally, strokes are commonly missed on CT when small, when hyperacute (within six hours), or when located in specific areas such as the brainstem or cerebellum. Overall, multiple studies show that CT scans have a relatively low sensitivity for ischemic strokes and should not be used to rule out disease exclusively [3]. CT, however, is useful when excluding other causes of disease or hemorrhage, such as spinal stroke, hemorrhagic neoplasms, encephalitis, multiple sclerosis, postictal paresis, hypertensive encephalopathy, and psychiatric diseases [11]. 

Although more expensive and time-consuming, MRI, particularly diffusion-weighted imaging, is more sensitive and specific than CT and can detect an acute ischemic stroke, even sometimes within minutes [12]. Persistent or worsening symptoms, even in the context of negative initial CT scans like in this case, should heighten clinical suspicion for ischemia. This warrants advanced imaging techniques like MRI to ensure accurate diagnosis and timely intervention. In this case, the patient’s persistent symptoms despite negative CT findings prompted further investigation with MRI. The MRI subsequently revealed the presence of an infarct, affirming the initial clinical suspicion of a cerebrovascular event that was not captured by the earlier CT scans. MRI, with its higher resolution and sensitivity to early ischemic changes, is crucial for diagnosing strokes that present with subtle symptoms. The ability of MRI to detect various imaging findings can help detect the mechanism of stroke, impacting prognosis and guiding best treatment options for patients. ​​Lesion mismatch profiles on MRI can also aid in evaluating the risks and benefits of thrombolysis by indicating the amount of salvageable tissue and the age of ischemic lesions [7]. Thus, for multiple reasons, MRI is imperative when diagnosing and assessing stroke symptoms, and treatment and can lead to better prognosis and patient outcomes.

The lack of facial involvement and other focal neurological deficits in this patient highlights the diagnostic challenge of thalamic strokes, which can exhibit significant variability in sensory symptoms due to the involvement of different thalamic nuclei. Unlike more classic stroke presentations, lateral thalamic strokes often manifest with isolated sensory deficits, such as numbness or paresthesia, which can vary widely in distribution and intensity. This variability can complicate early diagnosis, as the symptoms may not align with common stroke patterns. In this case, the absence of more typical motor deficits or cranial nerve involvement may have contributed to the initial difficulty in recognizing the stroke in the ED. Acute ischemic strokes are missed in approximately 9%-14% of patients who present to the ED, especially in patients presenting with nonspecific complaints or atypical symptoms such as dizziness, nausea/vomiting, or altered levels of consciousness [13]. Clinicians should maintain a high index of suspicion for ischemic stroke in patients presenting with isolated sensory symptoms, as these can sometimes precede or occur independently of more common motor deficits [4].

Although the neurologist ultimately identified the ischemic stroke and initiated appropriate therapy, the variability in sensory loss in thalamic strokes could create ambiguity in the early stages of the assessment, potentially leading to a delayed or missed diagnosis. While dual antiplatelet therapy was started appropriately after imaging confirmed the stroke, this case underscores the importance of recognizing that sensory variability, especially in thalamic strokes, can obscure the diagnosis. Providers should consider advanced imaging early in cases with atypical sensory presentations to avoid missing ischemic events, even when common stroke symptoms are not present.

Timely and accurate evaluation of stroke symptoms can greatly impact outcomes due to continuous neuronal death occurring from ischemia. As more time elapses, patients are at greater risk of permanent deficits and even death dependent on the area and size of the lesion [14]. According to the American Heart Association, every one-hour delay in the hospital resulted in 11 months of healthy life lost [15]. For patients presenting with persistent or unexplained neurological symptoms, early utilization of MRI can be crucial, especially after negative initial CT scans. This approach can prevent diagnostic delays and facilitate prompt, appropriate treatment. 

This case report demonstrates the critical role of MRI in the diagnosis of ischemic stroke, particularly when initial CT scans are negative and symptoms persist. It underscores the need for a thorough diagnostic approach in patients with atypical stroke presentations and the importance of advanced imaging techniques in ensuring accurate and timely diagnosis.

## Conclusions

CVAs and other ischemic events of the brain may present in atypical ways that require detailed evaluation. Thalamic strokes further present with difficulty due to the varying nuclei that can be impacted, creating a wide range of symptom variations. We present a 51-year-old male who arrived at the ED demonstrating an atypical pattern of an ischemic event that was discovered on consequent MRI following two negative CTs. Through this case, we are shown the demand for more advanced imaging studies, such as MRI, when symptoms of a CVA are persistent following a negative CT. The novelty of a patient presenting with pure hemiparesthesia, without facial involvement or other focal neurological deficits, demonstrates how neurological cases may present in uncommon ways. This report focuses on a unique presentation of a CVA with appropriate decision-making to successfully manage the patient promptly and effectively. Our findings bring attention to the importance of timely clinical decision-making to ensure that correct treatment is delivered, avoiding further brain damage and improving patient outcomes in cases of atypical isolated thalamic strokes.
